# Effectiveness and students’ perception of tutor-guided AI navigation in undergraduate medical education in Sri Lanka: a quasi-experimental study

**DOI:** 10.1186/s12909-026-09467-2

**Published:** 2026-05-18

**Authors:** P. J. S. Randombage, M. Y. S. Ahamed, T. D. Kulathunga, H. Wickramasekara

**Affiliations:** 1 Department of Obstetrics and Gynaecology, Faculty of Medicine, University of Kelaniya, Ragama, Sri Lanka; 2 Department of Medical Education, Faculty of Medicine, University of Kelaniya, Ragama, Sri Lanka

**Keywords:** Artificial intelligence, Generative artificial intelligence, Education, medical, Students, medical

## Abstract

**Background:**

The integration of Artificial Intelligence (AI), particularly Large Language Models (LLMs) such as ChatGPT, into undergraduate medical education offers new opportunities for personalized learning. However, concerns remain regarding content accuracy, possible over-reliance and limited critical engagement when AI tools are used without guidance. Tutor-guided AI navigation has been proposed as a structured approach combining AI accessibility with educator oversight. This study aimed to evaluate the effectiveness and perception of tutor-guided AI navigation among medical undergraduates.

**Methods:**

The study was conducted among 87 final-year medical students at the Faculty of Medicine, University of Kelaniya, Sri Lanka. A quasi-experimental pre-test/post-test design compared knowledge gains following tutor-guided versus self-directed ChatGPT-assisted learning. A cross-sectional survey assessing three domains-Perception of Learning Experience, AI Usability and Confidence, Satisfaction and Overall Impression- was administered immediately after the session. Responses were measured using a five-point Likert scale. Internal consistency was evaluated with Cronbach’s alpha and descriptive statistics were analysed using SPSS.

**Results:**

Baseline pre-test scores were comparable between groups (W = 670.5, *p* = 0.153). Both groups improved significantly from pre- to post-test (*p* < 0.001), with median scores increasing from 4 to 7. Knowledge gain between groups was not statistically significant (Welch’s t = 1.07, *p* = 0.144), although the effect size (Hedges’ g = 0.36) suggested a small-to-moderate advantage for tutor guidance. In the perception survey, reliability was high (Cronbach’s alpha > 0.86). Students rated ease of use (4.19 ± 1.07), tutor-guided AI experience (4.03 ± 1.13), satisfaction (3.91 ± 0.94) and willingness to recommend (4.01 ± 0.97) favourably, while trust in AI accuracy was moderate (3.27 ± 0.91).

**Conclusion:**

Tutor-guided AI-assisted learning was well accepted by students and provided a positive, structured learning experience. Although it did not produce a statistically significant advantage in immediate knowledge gain over self-directed AI use, it was associated with favourable perceptions of usability, satisfaction and recommendation. These findings support the structured integration of tutor-guided AI into undergraduate medical education while maintaining educator oversight and critical appraisal of AI-generated content.

**Supplementary Information:**

The online version contains supplementary material available at 10.1186/s12909-026-09467-2.

## Background

Artificial Intelligence (AI) technologies are reshaping the educational landscape, particularly in the field of medical education. Large Language Models (LLMs) like ChatGPT have been popularized for their potential to make learning experiences more personalised, interactive and available on demand. These AI systems can integrate vast amounts of information, generate comprehensive explanations and simulate clinical reasoning scenarios, making them smart learning partners [1–3].

Recent research indicates that AI technologies can assist medical students in gaining and applying information in multiple areas, such as clinical reasoning, anatomy and diagnostic interpretation [4, 5]. LLMs like ChatGPT are particularly interesting due to their conversational interface, which allows for repeated questioning and explanation, mirroring the Socratic way of learning [6]. Also, their 24/7 availability can make it easier for self-paced and remote learners to use them [7].

But even though they seem promising, there are still concerns regarding the safety and reliability of unguided AI use for learning. LLMs are likely to generate inaccurate or misleading information and they often do it with a high degree of confidence. This is called “AI hallucination” [8]. This risk is especially concerning in medicine, where accuracy and clinical judgement are of the utmost importance. Also, if students don’t get the right help, they can rely too much on AI-generated outputs, which could lead to shallow learning, less critical thinking and less growth in their clinical reasoning skills [9].

To overcome these issues, the idea of AI navigation with the help of a tutor has been proposed. This teaching method combines the benefits of AI with the guidance and support that teachers give. Tutors can help students create good prompts, understand AI responses, check the accuracy of content and have thoughtful discussions [10, 11].

Structured facilitation may improve cognitive engagement, encourage metacognition and reduce the chances of passive learning or misinformation. This approach is grounded in Scaffolded Learning Theory, whereby tutor guidance supports learners progressively, gradually building independence as competence grows [12]. Additionally, Cognitive Load Theory suggests that structured facilitation reduces extraneous cognitive load, allowing learners to direct cognitive resources toward meaningful knowledge acquisition rather than navigating unfamiliar AI-generated content unaided [13].

New research backs up the idea that scaffolded AI learning is useful. Research conducted by Noroozi et al. and Jin et al. indicates that guided AI interactions enhance information retention, increase learner satisfaction and improve problem-solving skills in comparison to self-directed AI usage [10, 14]. Moreover, ethical standards are progressively promoting human supervision in the implementation of AI within educational contexts to prevent bias and excessive dependence [11, 15].

Even with these changes, there isn’t much real-world data on how well tutor-guided AI models work in undergraduate medical education, especially in low- and middle-income countries (LMICs). To responsibly and effectively add AI technologies to medical curriculum, it’s important to understand how students feel about them, how easy they are to use and how they affect learning.

This study evaluated the effectiveness of tutor-guided AI navigation in undergraduate learning across three domains: perception of the learning experience, usability and confidence with AI and satisfaction and overall impression.

## Methods

### Study design

This research was conducted in two parts:

- Quasi-experimental study: A pre-test/post-test design compared knowledge acquisition in students who underwent the tutor-guided AI session versus those who used ChatGPT independently (intervention: *n* = 59; control: *n* = 28).
- Cross-sectional survey: Assessed students’ perception after the tutor-guided learning session.

A convenience sampling method was employed based on rotation schedule, session timing, availability and willingness to participate.

### Study setting and participants

The study was conducted at the Faculty of Medicine, University of Kelaniya, Sri Lanka among final-year undergraduate medical students. Participation was voluntary and informed consent was obtained from all participants. The study was approved by the institutional Ethics Review Committee (Ref No: P/116/06/2025).

### Intervention

The intervention group participated in a 90-minute session that integrated ChatGPT-based learning facilitated by a tutor. The tutor provided structured prompts aligned with key learning objectives in Obstetrics and Gynaecology and guided the students in interpreting and critiquing AI-generated content. The control group used ChatGPT independently to explore the same content.

### Assessments

#### Knowledge tests 

Students in both groups completed identical pre- and post-session MCQs.

A validated Single Best Answer (SBA) knowledge test was developed based on authoritative clinical guidelines [e.g. Sri Lanka College of Obstetricians & Gynaecologists (SLCOG), National Institute for Health and Care Excellence (NICE) guideline on diabetes in pregnancy and local obstetric diabetes protocols].

Each knowledge test comprised 10 Single Best Answer (SBA) questions, with one mark awarded per correct response, giving a maximum score of 10.

#### Perception questionnaire 

A pre-defined, self-administered questionnaire comprising 5-point Likert scale items was used to evaluate the learning experience, usability, confidence using AI, trust in AI-generated information, overall satisfaction and likelihood of recommending the method.

### Statistical analysis

All statistical analyses were performed using SPSS version 26. The Wilcoxon rank-sum test assessed within-group changes, while Welch’s t-test compared the magnitude of knowledge gains between groups. Hedges’ g was used to estimate effect size. Reliability of perception items was evaluated using Cronbach’s alpha.

## Results

### Knowledge acquisition

**Fig. 1:**
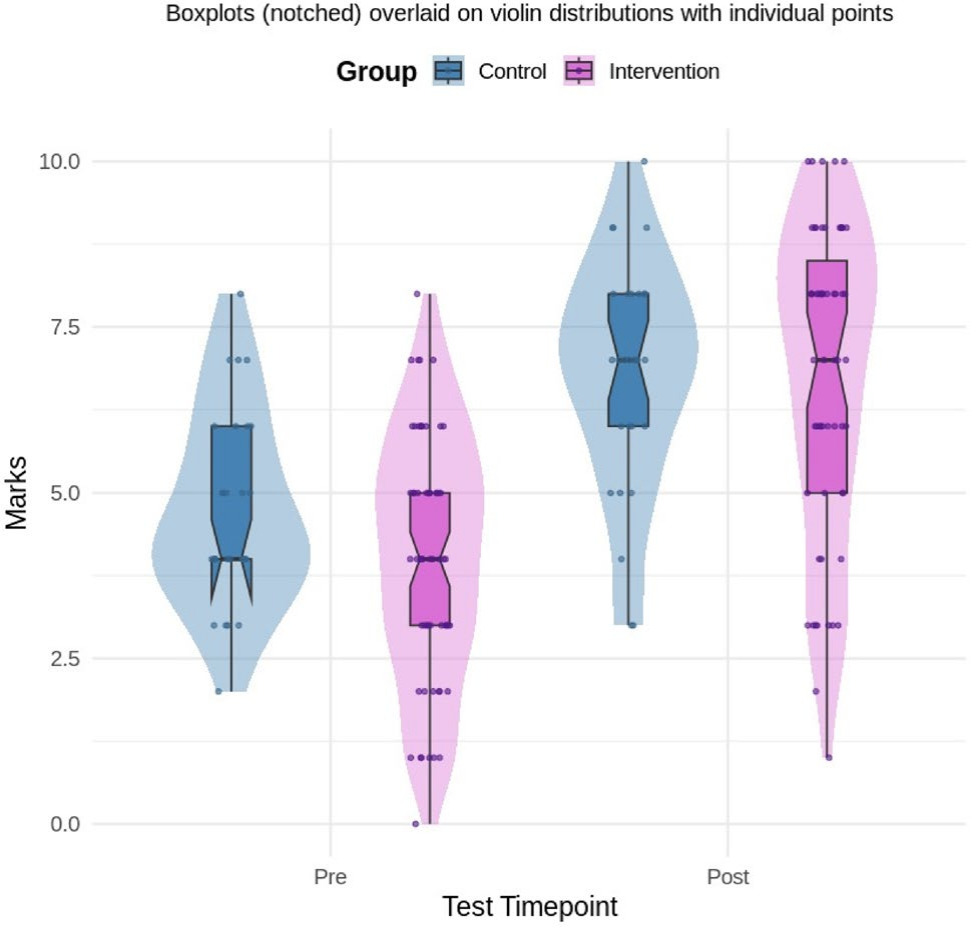
Pre and post test marks distribution by groups

The pre-test scores were statistically comparable between the two groups (W = 670.5, *p* = 0.153). Both groups demonstrated significant improvement from pre-test to post-test (*p* < 0.001), with median scores increasing from 4 to 7. The difference in the degree of improvement between the groups was not statistically significant (Welch’s t = 1.07, *p* = 0.144); however, the effect size (Hedges’ g = 0.36) indicated a small-to-moderate benefit for the tutor-guided group, suggesting a potentially meaningful educational benefit of tutor-guided AI-assisted learning (Fig. 1).

### Students’ perception

**Fig. 2:**
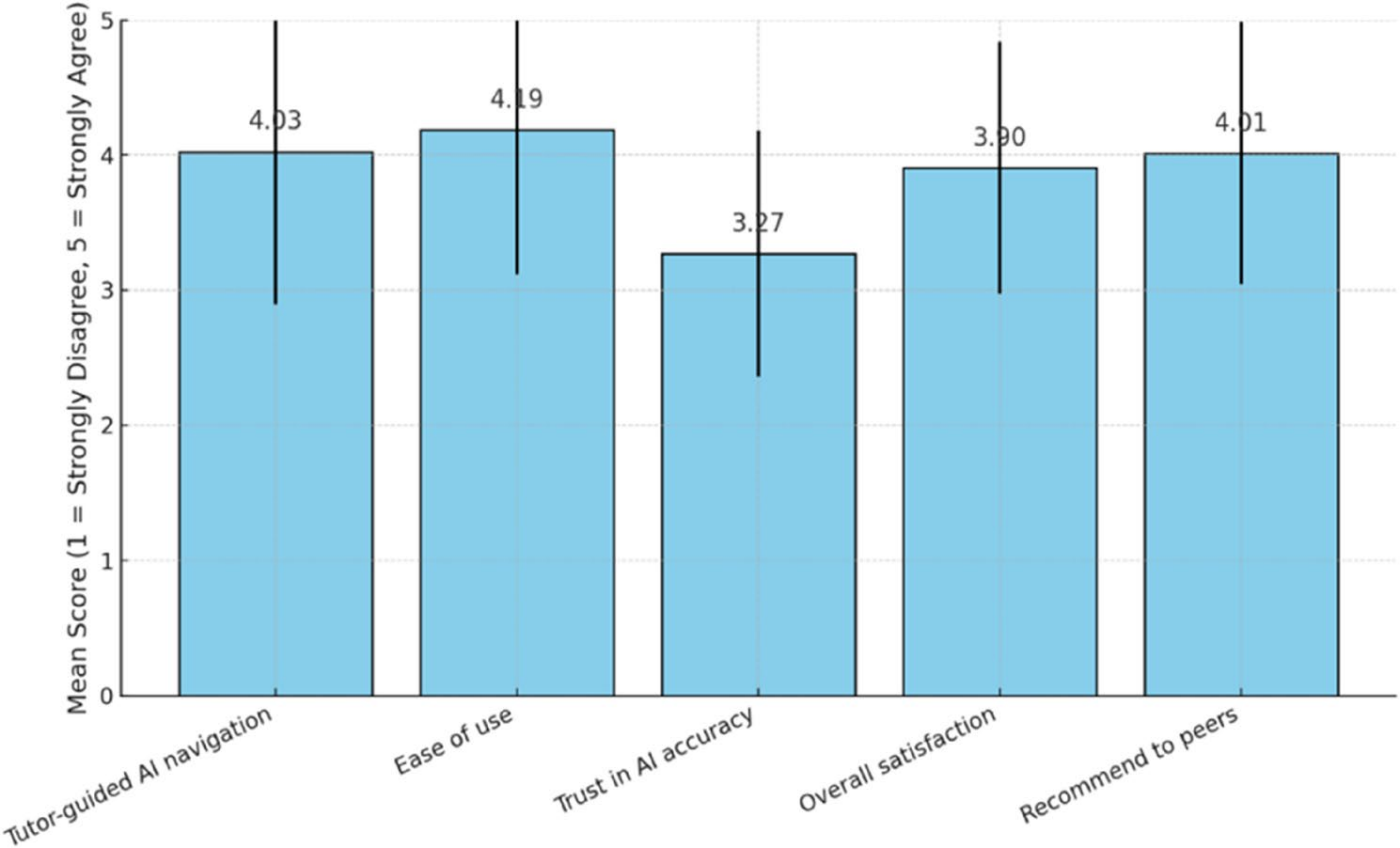
Students’ perception of tutor guided AI assisted learning

The perception survey, completed by 80 students, showed high reliability across all domains (α > 0.86). Students reported high levels of satisfaction and ease of use, with mean scores as depicted in Table 1; Fig. 2.

**Table 1:** Students’ perception of tutor guided AI assisted learning

Response	Mean Score ± SD
Ease of use	4.19 ± 1.07
Experience of tutor-guided AI navigation	4.03 ± 1.13
Trust in AI accuracy	3.27 ± 0.91
Overall satisfaction	3.91 ± 0.94
Willingness to recommend	4.01 ± 0.97

Students particularly appreciated the structured nature of the sessions and the opportunity to discuss AI output critically with a tutor.

## Discussion

### Interpretation of results

The results demonstrate that both tutor-guided and self-directed AI use can significantly improve short-term knowledge acquisition. While the difference between groups was not statistically significant, the structured guidance offered in the intervention appeared to enhance learning engagement and perceived value.

### Educational value of tutor-guided AI navigation

Tutor-guided AI learning may bridge the gap between traditional pedagogy and emerging educational technologies. The presence of a tutor allowed for real-time clarification of misconceptions, critical appraisal of AI-generated information and reinforcement of learning through dialogue. This hybrid approach is especially relevant in settings where students may lack the digital literacy or confidence to engage meaningfully with AI tools. Guided use of AI fosters greater confidence among students in critically evaluating and applying AI-generated responses within academic and clinical contexts [16–18].

This is consistent with Scaffolded Learning Theory, which posits that learner performance is enhanced when guidance is calibrated to individual competence levels and progressively withdrawn as mastery develops [12]. Furthermore, from a Cognitive Load Theory perspective, tutor-guided AI navigation may reduce the extraneous cognitive demands of unstructured AI interaction, enabling students to engage more deeply with clinical content and reasoning [13].

Moreover, the positive perception of this model among students underscores its potential scalability. As AI tools become increasingly integrated into educational platforms, faculty training and structured implementation will be key to maximizing their impact [19, 20].

### Implications for undergraduate medical education

The findings suggest that AI tools such as ChatGPT can be effectively integrated into undergraduate medical education without compromising learning outcomes. However, the added value of tutor guidance appears to lie not in immediate knowledge gains but in enhancing learning quality, engagement and professional formation. This supports the view that AI functions most effectively as a complementary educational tool, preserving the central role of human instruction while enriching the learning experience [20, 21].

This has important implications for curriculum design. Rather than viewing AI as a replacement for teaching, educational institutions should consider AI as a co-learning tool. Tutor-guided AI activities can promote deeper learning by encouraging questioning, contextualisation and reflection skills essential for clinical practice. When structured appropriately, AI-supported learning has been shown to achieve outcomes comparable to expert-led instruction, reinforcing its potential as a scalable adjunct in undergraduate training [22].

A key strength of tutor-guided AI navigation is its potential scalability in resource-limited settings where faculty shortages are prevalent. Tutors can configure Large Language Models with structured prompts, curated content and defined learning objectives, which can subsequently be shared with students for independent use. Once established, students who have participated in structured sessions acquire the foundational competencies and prompt literacy necessary to navigate AI tools independently and critically thereafter, effectively nullifying the requirement for repeated tutor involvement and continuous oversight. This model is therefore particularly relevant for institutions facing faculty shortages, as the initial investment in structured tutor guidance could potentially yield a replicable, self-sustaining learning framework accessible to large student groups.

The findings align with previous studies by Noroozi et al. and Jin et al., which suggest that structured AI use can enhance learning engagement [10, 14]. Wadden and Seaborn have highlighted the ethical and pedagogical risks of unregulated AI use in education [8, 9]. This study contributes novel data from a South Asian LMIC context, providing valuable insights for resource-limited settings.

The following limitations have been identified. The sample was restricted to final-year medical students at a single institution and a single clinical topic within Obstetrics and Gynaecology, which limits the generalisability of findings to broader medical education contexts. This study employed convenience sampling with non-randomised group allocation, which may introduce selection bias. The study measured only immediate knowledge gain; long-term retention and application in clinical contexts were not assessed. Both the pre-test and post-test comprised identical questions; therefore, score improvement may partly reflect familiarity with test items rather than solely representing knowledge acquisition. Future studies should consider using parallel forms to mitigate this confounder.

## Conclusion

Tutor-guided AI navigation using ChatGPT is a viable, effective and well-received approach for medical education. Although it did not demonstrate a statistically significant advantage over self-directed AI use in immediate knowledge acquisition, it was associated with higher levels of student satisfaction, engagement and perceived educational value.

These findings suggest that the primary benefit of tutor-guided AI lies in enhancing engagement, confidence and reflective learning rather than solely improving short-term knowledge gain. The balanced level of trust demonstrated by students further emphasizes the importance of structured guidance to support critical appraisal and professional judgment in tutor guided AI navigation.

### Recommendations

This study recommends integrating tutor-guided AI into undergraduate medical curricula as a structured, blended learning approach rather than an unstructured, self-directed tool. Incorporating AI into sessions like case-based discussions, problem-based learning and formative assessments can align with curricular goals while fostering critical thinking, contextual understanding and reflective learning. This approach positions AI as a complementary educational resource that enhances learning depth and consistency without undermining established pedagogical frameworks.

Effective implementation of tutor-guided AI learning requires systematic faculty development. Educators must be equipped with competencies in AI prompt formulation, verification of AI-generated content and facilitation techniques that encourage analytical thinking and reflective discussion.

In parallel, institutions should establish clear ethical guidelines to govern the responsible use of AI in medical education. Such frameworks should address issues of academic integrity, appropriate reliance on AI-generated information and the necessity of human oversight in learning and assessment. To inform evidence-based curriculum reform, future research should adopt longitudinal designs to evaluate the impact of tutor-guided AI learning on knowledge retention, clinical reasoning and decision-making in authentic clinical environments. These investigations will be critical in determining the sustained educational value of guided AI integration and in shaping best practices for its scalable implementation.

Future studies should employ larger, multi-site, multi-cohort designs spanning diverse clinical topics and multiple stages of undergraduate and postgraduate training to improve generalisability and establish more robust evidence for tutor-guided AI navigation in medical education.

## Supplementary Information

Supplementary Material 1.

## Data Availability

The datasets generated and/or analysed during the current study are available from the corresponding author on reasonable request.

## Abbreviations

AI: Artificial Intelligence
LLMs: Large Language Models
LMICs: Low–and Middle–Income Countries
MCQs: Multiple Choice Questions
