# Supplementary material for: Effectiveness and students’ perception of tutor-guided AI navigation in undergraduate medical education in Sri Lanka: a quasi-experimental study
Source: BMC Med Educ. 2026 May 18;26:1104. doi: 10.1186/s12909-026-09467-2 (PMC13348555; doi:10.1186/s12909-026-09467-2)
Supplement: Supplementary file 1 — Supplementary Material 1. [file 12909_2026_9467_MOESM1_ESM.docx]

**Structured Questionnaire for Students**

**Section A: Demographic and Academic Information**

- 1. Age:
  2. Gender: ☐ Male ☐ Female ☐ Other
  3. Are you currently in the final year of the MBBS program? ☐ Yes ☐ No
  4. Have you previously used AI tools like ChatGPT for learning? ☐ Yes ☐ No
  5. What are the AI tools you have used before? …………………….……….

**Section B: Perception of Learning Experience (Likert Scale: 1 = Strongly Disagree, 5 = Strongly Agree)**

| **Statement** | **1** | **2** | **3** | **4** | **5** |
| --- | --- | --- | --- | --- | --- |
| **1. The session improved my understanding of the subject.** |  |  |  |  |  |
| **2. The learning method was more engaging than traditional face to face lectures.** |  |  |  |  |  |
| **3. I was able to apply critical thinking during the session.** |  |  |  |  |  |
| **4. The method supported my self-directed learning skills.** |  |  |  |  |  |
| **5. I would prefer this learning method in future modules.** |  |  |  |  |  |
| **6. The tutor guided AI navigation helped clarify complex topics effectively.** |  |  |  |  |  |

**Section C: Usability and Confidence with AI**

| **Statement** | **1** | **2** | **3** | **4** | **5** |
| --- | --- | --- | --- | --- | --- |
| **7. The AI tool (e.g., ChatGPT) was easy to use.** |  |  |  |  |  |
| **8. I trusted the accuracy of the information provided by the AI tool.** |  |  |  |  |  |
| **9. Tutor guidance helped me interpret AI outputs more reliably.** |  |  |  |  |  |
| **10. I felt confident making clinical judgments after using this method.** |  |  |  |  |  |

**Section D: Satisfaction and Overall Impression**

| **Statement** | **1** | **2** | **3** | **4** | **5** |
| --- | --- | --- | --- | --- | --- |
| **11. I was satisfied with the overall learning experience.** |  |  |  |  |  |
| **12. This method made me more reflective and analytical.** |  |  |  |  |  |
| **13. Tutor involvement enhanced the value of AI-generated learning.** |  |  |  |  |  |
| **14. I would recommend tutor-guided AI learning to peers.** |  |  |  |  |  |

**Section E: Open-ended Feedback**

1. What did you like most about the session?
2. What challenges did you face during the learning session?
3. Suggestions to improve AI integration in medical education:

# **SBA questions on** **hyperglycemia in pregnancy (GDM and pregestational DM)**

**SBA 1 – Long-term Offspring Outcome**

A 30-year-old woman with poorly controlled type 1 diabetes mellitus (HbA1c 10.2% at 8 weeks gestation) delivers a macrosomic male infant at 38 weeks. The neonate develops neonatal hypoglycemia and respiratory distress. At the 6-month follow-up, the child shows delayed weight gain and abnormal glucose metabolism.

# Which fetal programming effect is most strongly supported by current evidence as a long- term

# consequence of maternal hyperglycemia in utero?

1. Increased risk of childhood epilepsy
2. Permanent beta-cell destruction in the pancreas
3. Epigenetic modification predisposing to insulin resistance
4. Decreased hypothalamic-pituitary-adrenal axis sensitivity
5. Reduced linear growth in adolescence

**Correct Answer:** C. Epigenetic modification predisposing to insulin resistance

# **SBA 2 – Intrapartum Management Nuance**

A 34-year-old woman with gestational diabetes requiring basal-bolus insulin presents at

39+1 weeks in early labor. Her most recent capillary glucose was 5.9 mmol/L. She reports a tendency toward hypoglycemia during late pregnancy. Her growth scan 3 days ago showed an EFW of 3.9 kg and normal Dopplers.

# What is the most appropriate glucose target during intrapartum management according to

# RCOG/NICE guidance?

1. Maintain maternal glucose <4.0 mmol/L to minimize fetal hyperinsulinemia
2. Maintain glucose between 3.5–5.0 mmol/L to prevent neonatal hypoglycemia
3. Target glucose levels <6.7 mmol/L using hourly monitoring
4. Withhold glucose monitoring unless the patient becomes symptomatic
5. Discontinue insulin therapy intrapartum if baseline glucose is <6.0 mmol/L

**Correct Answer:** C. Target glucose levels <6.7 mmol/L using hourly monitoring

# **SBA 3 – Pharmacology and Preconception Care**

A 36-year-old woman with type 2 diabetes, BMI 38, is planning pregnancy. She is on metformin, glargine insulin, losartan, and rosuvastatin. Her HbA1c is 7.1%. She has microalbuminuria but normal creatinine.

# What is the most appropriate adjustment to her pharmacological regimen before attempting

# conception?

1. Switch rosuvastatin to fenofibrate and continue losartan
2. Substitute glargine with NPH insulin due to teratogenic risk
3. Discontinue losartan and start labetalol before conception
4. Increase metformin dose to 2 g/day to improve insulin sensitivity
5. Continue all medications until fetal cardiac activity is confirmed

**Correct Answer:** C. Discontinue losartan and start labetalol before conception

(*Note: ACEi/ARBs are contraindicated pre-conception and during pregnancy due to risk of fetal renal dysgenesis and oligohydramnios.*)

# **SBA 4 – Diagnostic Dilemma in Recurrent GDM**

A 29-year-old woman had diet-controlled GDM in her first pregnancy, and insulin-controlled GDM in her second. She presents at 10 weeks gestation during her third pregnancy. Fasting glucose is 5.1 mmol/L; random glucose 8.8 mmol/L; HbA1c is 6.3%. BMI is 34.

# Which investigation is most appropriate to determine the underlying pathophysiology of her

# hyperglycemia at this stage?

1. Oral glucose tolerance test at 24 weeks
2. Fasting insulin and C-peptide levels
3. HOMA-IR calculation for insulin resistance
4. Anti-GAD and anti-IA2 autoantibodies
5. Continuous glucose monitoring for 1 week

**Correct Answer:** D. Anti-GAD and anti-IA2 autoantibodies

(*To rule out latent autoimmune diabetes of adults [LADA] or early type 1 DM.*)

# **SBA 5 – Complex Fetal Surveillance**

A 40-year-old woman with longstanding poorly controlled type 2 diabetes (HbA1c 8.5% at 28 weeks) and early-stage diabetic nephropathy is under combined care. Serial growth scans

show stable estimated fetal weight at the 10th percentile. UA Doppler and MCA Doppler are within normal limits.

# What is the most appropriate next step in fetal surveillance at 34 weeks?

1. Initiate twice-weekly biophysical profile scoring
2. Schedule elective delivery at 36 weeks without further testing
3. Perform weekly umbilical artery Doppler and amniotic fluid assessment
4. Add uterine artery Doppler to assess placental insufficiency
5. Plan fetal MRI to evaluate for CNS malformations

**Correct Answer:** C. Perform weekly umbilical artery Doppler and amniotic fluid assessment (*FGR in diabetic mothers with nephropathy requires careful monitoring of placental function to guide timing of delivery.*)

# **SBA 6 – Metabolic Programming and Maternal Obesity**

A 32-year-old woman with class II obesity (BMI 37) and diet-controlled gestational diabetes gives birth at 39 weeks. The infant has a birth weight of 4.2 kg and mild neonatal

hypoglycemia. At the postnatal visit, the mother expresses concern about her child’s long- term metabolic health.

Which factor most strongly amplifies the adverse intrauterine metabolic programming effects of maternal hyperglycemia?

1. Excess maternal gestational weight gain
2. First-trimester folate supplementation
3. Tight glycemic control during labor
4. Use of metformin as first-line therapy
5. Fetal sex (male fetus)

# Correct Answer: A. Excess maternal gestational weight gain

(*Excess weight gain in GDM amplifies the risk of childhood obesity and metabolic dysfunction independent of glycemic control.*)

# **SBA 7 – Immunopathogenesis in Pregestational Diabetes**

A 26-year-old woman with newly diagnosed diabetes during early pregnancy has a fasting glucose of 6.1 mmol/L, positive anti-GAD antibodies, and low C-peptide levels. BMI is 23 kg/m². She is diagnosed with latent autoimmune diabetes in adults (LADA).

What is the most appropriate management implication of this immunologic profile in pregnancy?

1. Insulin should be initiated only if postprandial glucose exceeds 7.8 mmol/L
2. Immunosuppressive therapy is indicated to delay beta-cell destruction
3. She should be monitored closely for rapid progression to insulin dependence
4. Metformin monotherapy is safe and preferred throughout pregnancy
5. Pancreatic autoantibodies resolve spontaneously during pregnancy

# Correct Answer: C. She should be monitored closely for rapid progression to insulin dependence

(*LADA may appear as mild diabetes early but often progresses rapidly during the stress of pregnancy.*)

# **SBA 8 – Neonatal Outcomes and Glycemic Variability**

A 38-year-old woman with type 1 diabetes has good average glycemic control (HbA1c 6.4%) but significant glycemic variability with frequent nocturnal hypoglycemia and postprandial spikes. She delivers a 3.5 kg infant at term.

Which neonatal complication is **most strongly associated** with maternal glycemic variability despite overall HbA1c being acceptable?

1. Neural tube defects
2. Polycythemia and hyperbilirubinemia
3. Pulmonary hypoplasia
4. Fetal alcohol syndrome–like features
5. Trisomy 13

# Correct Answer: B. Polycythemia and hyperbilirubinemia

(*Wide glycemic swings are linked to fetal hyperinsulinemia, which increases red cell mass and postnatal bilirubin.*)

# **SBA 9 – Metformin Failure in Gestational Diabetes**

A 35-year-old woman with newly diagnosed gestational diabetes at 28 weeks gestation is started on metformin. Despite escalation to the maximum tolerated dose, her fasting and postprandial glucose levels remain above target. Her BMI is 32 kg/m². She is adherent to both medication and dietary recommendations.

Which factor is **most likely responsible** for her suboptimal glycemic response to metformin during pregnancy?

1. Physiological insulin resistance due to placental hormones
2. Delayed gastric emptying secondary to pregnancy hormones
3. Impaired renal clearance of metformin in late gestation
4. Glucagon-like peptide 1 (GLP-1) deficiency
5. Overestimation of carbohydrate intake by dietary logs

# Correct Answer: A. Physiological insulin resistance due to placental hormones

(*As pregnancy progresses, increased levels of placental hormones such as human placental lactogen and cortisol contribute to insulin resistance, which may overwhelm the effect of metformin, necessitating insulin initiation.*)

# **SBA 10 – Retinopathy and Glycemic Optimization**

A 30-year-old woman with type 1 diabetes for 15 years becomes pregnant. Her HbA1c has dropped from 9.2% to 6.3% within 10 weeks following aggressive glycemic optimization. She now reports blurred vision at 14 weeks.

What is the most likely cause of her symptoms?

1. Normal pregnancy-related visual changes
2. Rapid worsening of diabetic retinopathy
3. Metformin-induced retinal edema
4. Insulin allergy-related vasculitis
5. Posterior reversible encephalopathy syndrome (PRES)

# Correct Answer: B. Rapid worsening of diabetic retinopathy

(*Sudden glycemic improvement can lead to progression of retinopathy in pre-existing diabetes.*)

# **Structured AI Prompts for SBA Learning on Hyperglycemia in Pregnancy**

For each prompt, the tutor will:

- - Explain the learning objective.
  - Provide the prompt to be entered into ChatGPT
  - Facilitate discussion about the AI's response.
  - Encourage critical appraisal of content accuracy and clinical relevance.
  - Conduct a reflective discussion afterward.

# Structured AI Prompts for SBA Learning on Hyperglycemia in Pregnancy

| **QUESTION NO.** | **LEARNING FOCUS** | **CHATGPT PROMPT** |
| --- | --- | --- |
| **SBA 1** | Fetal programming and long-term metabolic outcomes | “What are the long-term metabolic effects on offspring exposed to maternal hyperglycemia during pregnancy?” |
| **SBA 2** | Intrapartum glucose management in GDM | “What are the NICE and RCOG recommendations for intrapartum glucose targets in women with gestational diabetes on insulin?” |
| **SBA 3** | Preconception pharmacology and contraindicated drugs | “Which medications for type 2 diabetes and comorbidities must be adjusted or stopped before pregnancy and why?” |
| **SBA 4** | Autoimmune diabetes in pregnancy | “How do we differentiate gestational diabetes from latent autoimmune diabetes in adults (LADA) during early pregnancy?” |
| **SBA 5** | Fetal surveillance in diabetic nephropathy | “What is the recommended fetal monitoring plan in pregnant women with diabetes and fetal growth restriction?” |
| **SBA 6** | Maternal obesity and fetal metabolic programming | “How does excessive maternal weight gain during GDM affect the long-term health of the child?” |
| **SBA 7** | LADA and insulin progression | “What is LADA in pregnancy, and how does it differ from type 1 and type 2 diabetes in management?” |

| **SBA 8** | Glycemic variability and neonatal complications | “How does maternal glycemic variability affect fetal outcomes despite normal HbA1c?” |
| --- | --- | --- |
| **SBA 9** | Metformin failure in GDM | “Why might metformin fail to control glucose in late pregnancy despite compliance?” |
| **SBA 10** | Rapid glycemic correction and retinopathy | “How does rapid improvement in blood glucose affect diabetic retinopathy during early pregnancy?” |
|  |  |  |

**Tutor Facilitation Notes:**

- - After each prompt, students will be encouraged to critically evaluate the AI's response using up-to-date clinical guidelines (e.g., NICE, RCOG).
  - Tutors will highlight any limitations or errors in AI responses and clarify correct clinical concepts.
  - Students will be asked to summarize key takeaways and the tutor can guide discussion questions, such as:
    - “How would this apply in a Sri Lankan clinical context?”
    - “Is this consistent with local or international protocols?”
    - “What other differential diagnoses or factors should be considered?”
